# Response of upper tropospheric water vapor to global warming and ENSO

**DOI:** 10.1038/s41598-024-56639-5

**Published:** 2024-03-12

**Authors:** Li Li, Zhiping Chen, Bingkun Wang, Jiao Fan, Tieding Lu, Kaiyun Lv

**Affiliations:** 1 School of Surveying and Geoinformation Engineering, East China University of Technology, Nanchang, 330013 China; 2 Key Laboratory of Mine Environmental Monitoring and Improving around Poyang Lake of Ministry of Natural Resources, East China University of Technology, Nanchang, 330013 China; 3 State Key Laboratory of Geodesy and Earth’s Dynamics, Innovation Academy for Precision Measurement Science and Technology, Chinese Academy of Sciences, Wuhan, 430077 China

**Keywords:** Climate sciences, Atmospheric science

## Abstract

The upper tropospheric water vapor is a key component of Earth's climate. Understanding variations in upper tropospheric water vapor and identifying its influencing factors is crucial for enhancing our comprehension of global climate change. While many studies have shown the impact of El Niño-Southern Oscillation (ENSO) and global warming on water vapor, how they affect the upper tropospheric water vapor remains unclear. Long-term, high-precision ERA5 specific humidity data from the European Centre for Medium-Range Weather Forecasts (ECMWF) provided the data foundation for this study. On this basis, we successfully obtained the patterns of global warming (Independent Component 1, IC1) and ENSO (Independent Component 2, IC2) by employing the strategy of independent component analysis (ICA) combined with non-parametric optimal dimension selection to investigate the upper tropospheric water vapor variations and responses to ENSO and global warming. The results indicate that global warming and ENSO are the primary factors contributing to water vapor variations in the upper troposphere, achieving the significant correlations of 0.87 and 0.61 with water vapor anomalies respectively. Together, they account for 86% of the global interannual variations in water vapor. Consistent with previous studies, our findings also find positive anomalies in upper tropospheric water vapor during El Niño years and negative anomalies during La Niña years. Moreover, the influence extent of ENSO on upper tropospheric water vapor varies with the changing seasons.

## Introduction

Water vapor in the mid-troposphere and upper-troposphere provides one of the most crucial amplifying feedbacks in the climate system^1–3^. Especially, the upper tropospheric water vapor is an important component of the Earth's climate and plays a crucial role in climate variability and weather changes^4,5^. It is also the most significant positive feedback factor in climate change^6^, exerting indirect influences on global climate change through interactions with clouds, aerosols, and tropospheric chemistry^4^. Specific humidity is one of the most commonly used indicators for describing water vapor changes. Studies have shown that minor changes of specific humidity in the upper atmosphere have a much more significant impact on the greenhouse effect compared to minor changes of water vapor in the lower atmosphere^7,8^. Palridge et al.^9^ investigated the trends in upper tropospheric humidity based on National Center for Environmental Prediction (NCEP) reanalysis. Their results suggest that water vapor feedback plays a crucial role at this altitude^9^. Therefore, understanding the characteristics of upper tropospheric water vapor variations and identifying factors that control these variations are crucial for comprehending global climate change. Vergados et al.^10^ noted that water vapor feedback is strongest at the 250 hPa pressure level in the upper troposphere. Therefore, this study chooses the representative 250 hPa pressure level in the upper troposphere to investigate variations in upper tropospheric water vapor. Additionally, changes in water vapor are controlled by the coupling of thermodynamics with atmospheric circulation. Therefore, the Clausius–Clapeyron equation in thermodynamics is also introduced in this paper. The Clausius–Clapeyron equation is a fundamental principle that governs changes in water vapor in the climate system. It provides a robust constraint on how the saturated water content varies with temperature^11^. Allan et al.^12^ indicated that the global water vapor responses can be explained by the thermodynamic amplification of upper tropospheric temperature changes and the Clausius–Clapeyron temperature dependence of saturated water vapor pressure. In accordance with this temperature dependence of the Clausius–Clapeyron equation, Allan et al. ^12^ noted that at higher altitudes in the troposphere, the global mean water vapor responds more strongly to temperature changes in simulations and reanalysis.

The most crucial feedback in the Earth’s climate system is the climate feedback induced by upper tropospheric water vapor^13^. Therefore, it is important to identify the climate factors influencing upper tropospheric water vapor. At present, research indicates that El Niño-Southern Oscillation (ENSO) is a crucial climate factor influencing variations in water vapor. Chen et al.^14^ crudely observed a relationship between humidity and ENSO based on General Circulation Models (GCMs). Trenberth et al.^15^ pointed out that the atmospheric water vapor content over the oceans is closely linked to ENSO events. Dessler et al.^1^ and Allan et al.^12^ quantitatively assessed the impact of ENSO on the upper troposphere using satellite and ground observations, reanalysis data, and climate simulations. The results indicate that ENSO is one of the factors influencing changes in upper tropospheric water vapor^1,12^. The findings of Lu et al. ^16^ suggest that the first and second empirical orthogonal function (EOF) modes of tropical cold point tropopause temperature anomalies are associated with ENSO activity. Saha et al.^4^ investigated the response of upper tropospheric water vapor to ENSO using European Centre for Medium Range Weather Forecasts Reanalysis v5 (ERA5) data. Their study indicates that the physical mechanisms linking ENSO to upper tropospheric water vapor are associated with deep convective changes in the central and eastern Pacific^4^. Their study also indicates that the impact of ENSO on interannual tropical water vapor anomalies is more pronounced in the upper troposphere. And the upper tropospheric water vapor exhibits positive anomalies during El Niño years, while it shows negative anomalies during La Niña years. Patel et al.^17^ investigated the long-term global tropospheric water vapor variations from 1980 to 2020. They showed that there are significant seasonal variations in tropospheric water vapor and that the water vapor variations in the tropics are strongly influenced by ENSO^17^. Wang et al.^18^ developed an eight-direction vector decomposition algorithm based on the EAR5 dataset to calculate the water vapor fluxes between the ocean and land. Their results showed that ENSO is a crucial factor influencing the anomalies of global ocean-land water vapor exchange^18^.

Global warming is also one of the key climate factors influencing water vapor variations. Lindzen ^19^ and Pierrehumbert ^20^ discussed the possible mechanism of global warming leading to the reduction of upper tropospheric humidity. On the other hand, Rind et al. ^21^ put forward the argument that global warming causes increased humidity in the upper troposphere. Dickinson et al.^22^ provided a definitive answer that water vapor feedback is positive in the context of global warming. Hall et al. ^23^ confirmed the opinion of Dickinson and their quantitative experiment demonstrates that the feedback of water vapor is significantly stronger in the context of global warming. Patel et al.^17^ stated that in the context of global warming, the increase in global surface temperatures leads to an increase in water vapor evaporation, ultimately resulting in an increase in atmospheric water vapor content. Hall et al.^23^ simulated the feedback mechanism of water vapor unaffected by the impact of global surface temperature. Their study suggests that global warming is the response to a steady, globally uniform forcing, and water vapor feedback will be stronger in the context of global warming^23^. Wang et al. ^18^ showed that global warming intensifies the water vapor transport between the ocean and land, and that the water vapor flux between oceans and land has increased by over 8% with rising temperatures.

While the above studies show the impact of ENSO and global warming on water vapor, how they affect the upper tropospheric water vapor remains unclear. In order to investigate the contributions of ENSO and global warming to the variations in upper tropospheric water vapor separately, it is essential to obtain the patterns of ENSO and global warming by employing the component extraction method. The presence of nonlinear features in the data can impact the selection of component extraction methods. When dealing with data containing non-linear features, employing linear methods may mix information between the different components, whereas non-linear methods will address this issue^24^. Existing studies indicate that not only do ENSO and global warming signals exhibit highly nonlinear characteristics, but the climate system itself is also a complex nonlinear model, whose climate time series exhibit non-Gaussian distribution features^25–27^. Independent Component Analysis (ICA) originates from the fields of information theory and statistics. It utilizes higher-order statistics to separate statistically independent components (ICs) from the non-Gaussian data^28,29^, which can effectively overcome the effects of nonlinear features. However, the classical ICA method suffers from the issue of uncertainty in the output dimension. Koch and Naito ^30^ emphasized that the quality of the components separated by ICA is affected by the output dimension. If the output dimension is too small, ICA will not find the largest non-Gaussian direction, leading to a loss of information. Conversely, an excessively large dimensionality may introduce noise. To solve this problem, this paper introduces the non-parametric optimal dimension selection method to determine the optimal output dimensions^30^, which utilizes the objective Akaike Information Criterion and data-driven criteria for selecting the optimal output dimensions.

While there have been many studies investigating the variations in upper tropospheric water vapor and their associated mechanisms and influencing factors, there are still some aspects that need to be further analyzed. To overcome the impact of non-linear characteristics of ENSO and the uncertainty in ICA dimensions selection, this study adopted the strategy of ICA combined with the non-parametric optimal dimension selection. ERA5 specific humidity data from the European Centre for Medium-Range Weather Forecasts (ECMWF) is a product of combining data assimilation with weather forecast modelling system, the accuracy of which relies on data assimilation methods and the stability and precision of observational systems. With the assistance of this methods and ERA5 specific humidity data, the ENSO and global warming patterns in the upper troposphere were successfully separated to further investigate the contributions of ENSO and global warming to the variations in upper tropospheric water vapor.

## Data and methods

The accuracy of ERA5 water vapor data relies on data assimilation methods and the stability and precision of observational systems. Microwave observations and infrared datasets (e.g., AMSU, HIRS) provide accurate estimates of water vapor variations in the upper troposphere^12,31,32^, while spectral or radio occultation data (e.g., AIRS) provides information on the distribution of specific humidity and relative humidity^33,34^. Since satellite data are important for determining water vapor changes, especially over the oceans, the scarcity of satellite data prior to the mid-1990s resulted in unrealistic trends in the ERA5 dataset^12,35^. The specific humidity data used in this article stems from the fifth generation of the ECMWF atmospheric reanalysis data, which is available in the European Union's Earth observation program ERA5 Copernicus Climate Data Store (https://cds.climate.copernicus.eu). ERA 5 specific humidity dataset is a product of combining data assimilation with weather forecast modelling system with high resolution and accuracy, which is crucial for studying the global water cycle, climate change, and extreme weather events^36^. The high spatial resolution (0.25° × 0.25°) and high temporal resolution (hourly) of ERA5 data make it possible to conduct precise studies of trends and variations in atmospheric water vapor^15^. A long-term time series of ERA5 specific humidity data spanning from January 1959 to December 2021 is selected in this paper to investigate the effects of ENSO and global warming on atmospheric water vapor in both temporal and spatial domains.

ENSO, driven by sea surface temperature variations in the central and eastern equatorial Pacific, is an important source of tropical Pacific climate anomalies. It not only has a significant impact on water vapor, but also is one of the most important factors contributing to global climate change. The Oceanic Niño Index (ONI) is the most suitable indicator for characterizing ENSO events^37^, and it is also one of the most commonly used indices for defining El Niño and La Niña events. Therefore, in order to quantitatively analyze the temporal and spatial effects of ENSO on water vapor, ONI (https://psl.noaa.gov/data/correlation/oni.data) from the National Oceanic and Atmospheric Administration (NOAA) Physical Sciences Laboratory is selected in this paper.

Global warming is a long-term phenomenon characterized by the widespread increase in the Earth’s surface temperature caused by greenhouse gases. It has profound impacts on ecosystems, economies, and societies^38,39^. The global mean surface temperature (GMST) is one of the most important indicators for measuring global warming and climate change^40^. Therefore, GMST (https://climate.nasa.gov/vital-signs/global-temperature/) is selected from (National Aeronautics and Space Administration) NASA Goddard Institute for Space Studies (GISS) to investigate the temporal and spatial response of water vapor to global warming.

**Figure 1:**
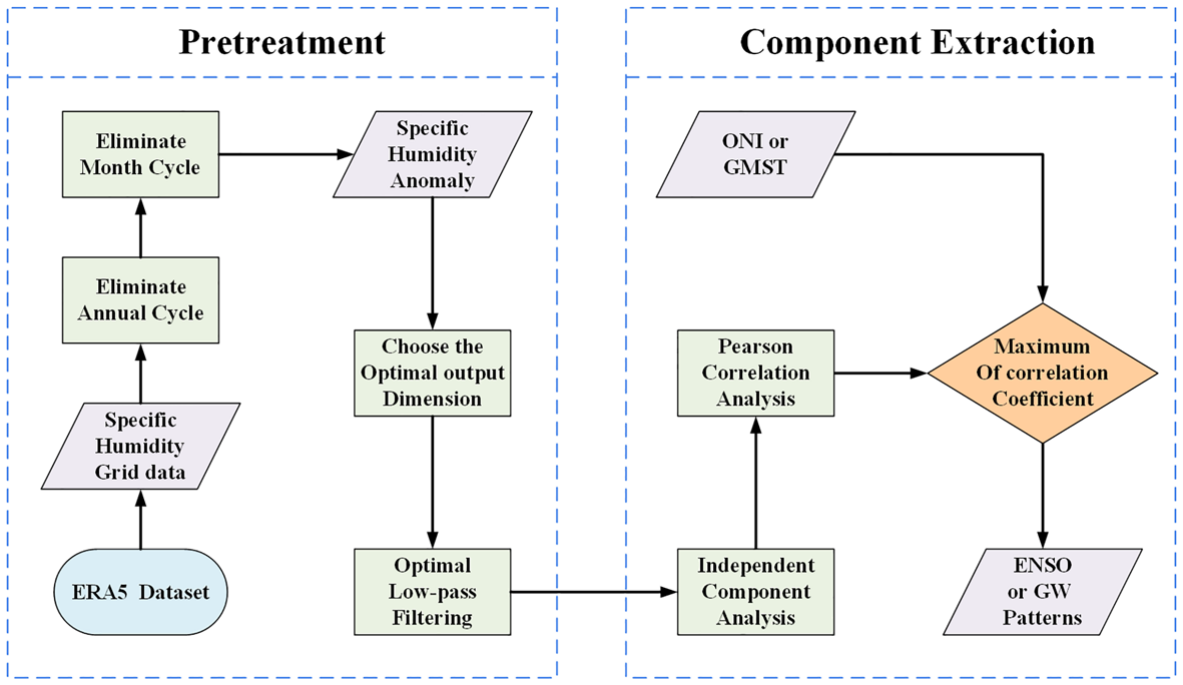
Processing flow chart of component extraction using the strategy of the optimal lowpass filtering combined with ICA.

Climate time series exhibit nonlinear characteristics. In order to overcome the impact of nonlinearity on the quality of component extraction, a new processing strategy of the optimal lowpass filtering combined with ICA is proposed for the first time. The complete process of the proposed method is illustrated in Fig. 1.

The process flow can be divided into two parts: pretreatment and component extraction. The pretreatment primarily consists of the following four steps. Firstly, the grid specific humidity data is obtained from the ERA5 dataset. Secondly, the specific humidity anomalies signals were obtained by eliminating the annual and month-to-month variations ^41^. In detail, the annual cycle was eliminated by subtracting the month-by-month climatic mean state. On the other hand, the 1-2-1 three point binomial filter was used to eliminate the monthly variability ^42^. Thirdly, the optimal output dimension is determined by the non-parametric method based on the specific humidity anomalies signals ^30,43^. The optimal output dimension is 2 according to the data used in this paper. Fourthly, in order to eliminate the noise from specific humidity anomalies signals, the optimal lowpass filtering was applied ^44^. However, due to the uncertainty of the optimal cut-off frequency of lowpass filtering, it is essential to compare the results with different cut-off frequencies to determine the optimal cut-off frequency.

According to the selected optimal output dimension, independent components can be estimated by ICA for all filtering cut-off frequencies. For each filtering cut-off frequency, Pearson correlation analysis was carried out between the ICs and ONI/GMST. The optimal cut-off frequency of the lowpass filter was determined when the maximum absolute value of the correlation coefficient was obtained. The time series of ICs which have the maximum absolute value of the correlation coefficient with ONI were regarded as ENSO pattern. Similarly, the time series of ICs which have the maximum absolute value of the correlation coefficient with GMST were regarded as global warming pattern.

## Results and discussion

### Characteristics of water vapor variations and the affecting factors

**Figure 2:**
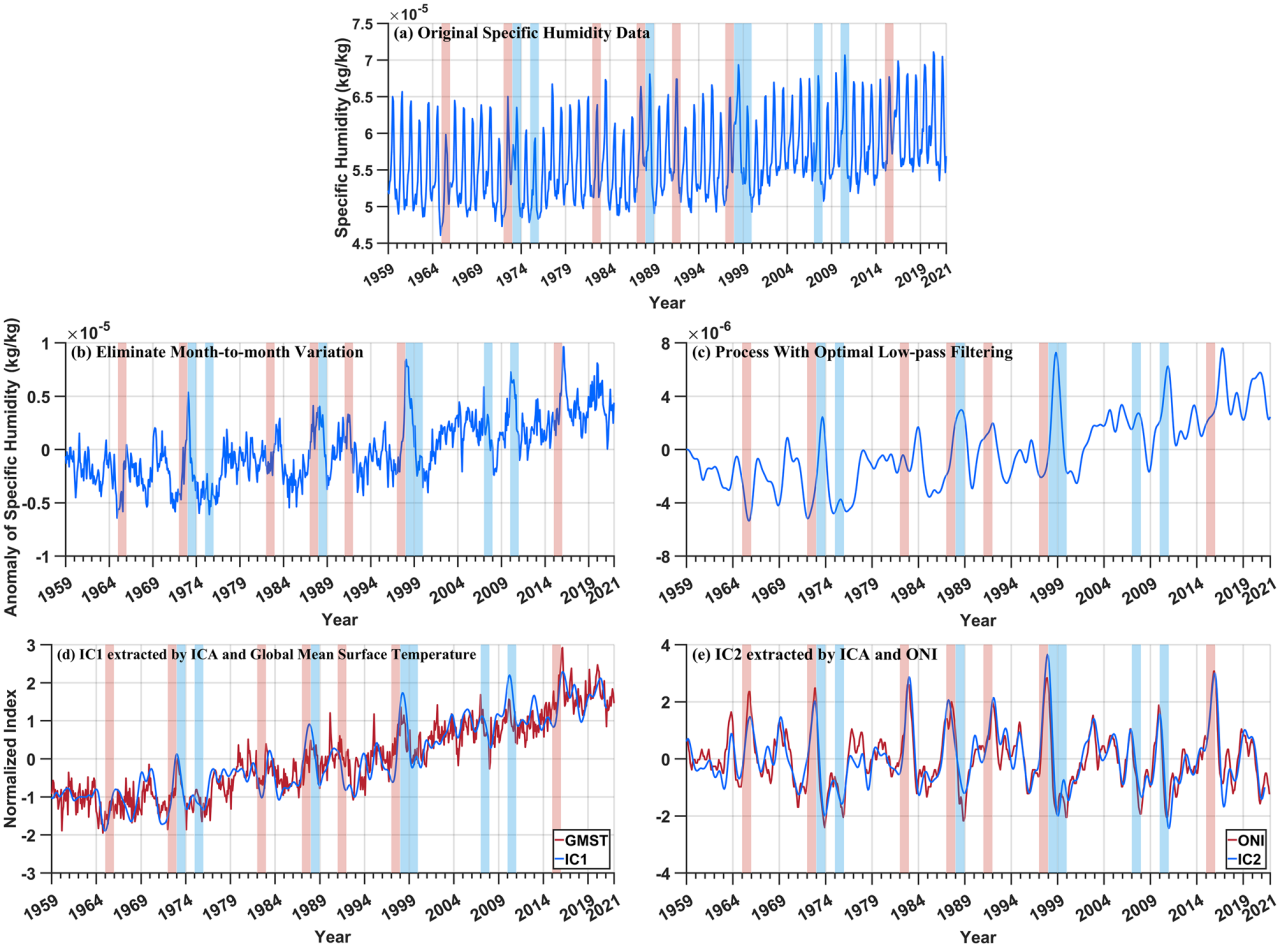
Processing flow chart of data preprocessing (**a–c**) and the component extraction of the affecting factors (**d**, **e**). (**a**) Global mean specific humidity; (**b**) Deducting the annual cyclic and monthly variations; (**c**) Processing of time series with optimal lowpass filter based on (**b**); (**d**) GMST and IC1 extracted by the strategy of ICA combined with the non-parametric optimal dimension selection; (**e**) ONI and IC2 extracted by the strategy of ICA combined with the non-parametric optimal dimension selection. the years of strong El Niño and La Niña events (the intensity of ENSO events covered in this paper is provided by the website: https://www.ggweather.com/enso/oni.htm) are highlighted in all the time series figure with light red and light blue backgrounds respectively.

To provide a clearer analysis of the characteristics of upper tropospheric water vapor variations and assess the factors influencing water vapor variations, the results of the time series after each processing step of the component extraction flow chart are presented here. Figure 2a displays the global mean specific humidity data, indicating a noticeable seasonal cycle in the specific humidity data. This is consistent with the findings of Patel et al.^17^, whose research investigated the long-term variations in global tropospheric water vapor and identified significant seasonal variations. This study also found that water vapor content is highest in summer and lowest in winter^17^. In addition, to investigate the factors influencing water vapor variations, this study used the strategy of ICA combined with the non-parametric optimal dimension selection to separate the corresponding patterns of global warming and ENSO from specific humidity anomalies. The results are shown in Fig. 2e and f, respectively.

From the time series of specific humidity anomalies after processing (Fig. 2b, c), it is evident that the time series exhibits a slow upward trend, which is consistent with Dessler et al.^1^, who also found a long-term upward trend in the upper tropospheric water vapor data. This may be associated with the increase in deep convection caused by global temperature rise^17^. From Fig. 2b and c, it can be observed that the majority of strong El Niño years correspond to the maximum values in the specific humidity anomalies time series, while strong La Niña years correspond to minimum values. This is consistent with the findings of Saha et al.^4^ and Tian et al.^45^, where the peak values of water vapor anomalies are associated with El Niño years, while the minimum values are associated with La Niña years. There may be a lag in the response of upper tropospheric water vapor to ENSO phenomenon, causing some ENSO years not having a good one-to-one correspondence with the maximum or minimum values. The lag phenomenon can be attributed to the fact that sea surface temperatures reach their peak in winter (DJF = December, January, and February) during ENSO events. Sea surface temperatures alters the pressure gradients over the Pacific Ocean, affecting trade winds and the Walker circulation. Subsequently, the Walker circulation determines the position of deep convection in the Pacific, causing the Kelvin wave to take 2–3 months to cross the Pacific. Additionally, the transition of convection also takes time. Only after the convection changes can water vapor be transported to the upper troposphere. Hence, there is a lag in the impact of ENSO on upper tropospheric water vapor, requiring several months to respond to ENSO^4^.

**Figure 3:**
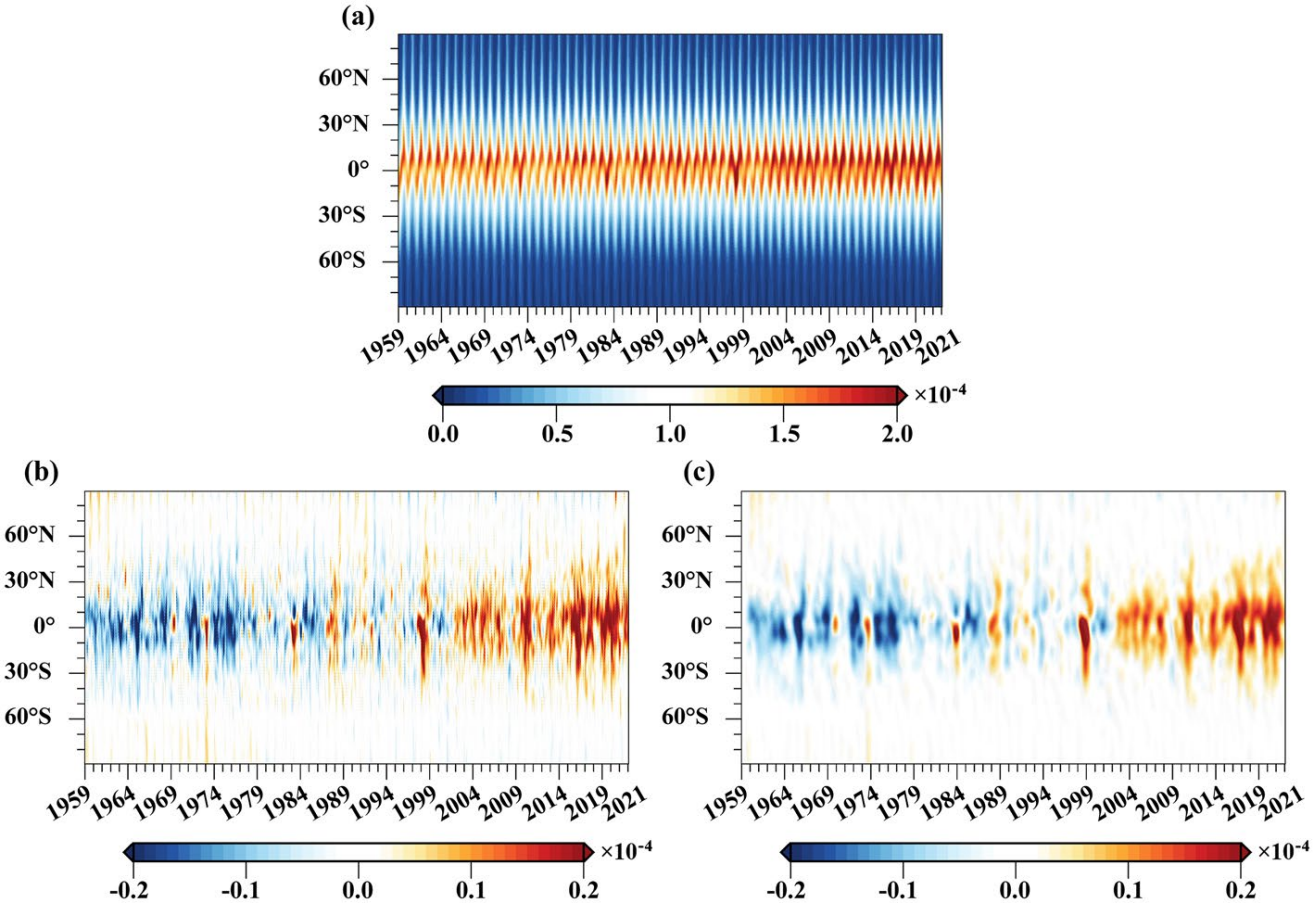
Latitude–time evolution of the global zonal mean specific humidity (Units: kg/kg). (**a**) Global specific humidity; (**b**) deducting the annual cyclic and monthly variation of the global specific humidity; (**c**) processing of time series with optimal lowpass filter based on (**b**).

The spatial distribution of water vapor flux exhibits clear latitude characteristics, with water vapor flux between oceans and land decreasing as latitude increases^18^. Additionally, under the influence of global warming, the spatial extent and the feedback of water vapor becomes stronger over time^23^. Figure 3a shows the latitude-time evolution of global specific humidity. It is evident that there is a significant annual cycle, and the influence range and intensity of water vapor increases over time. This result is consistent with the findings in Fig. 2a, which are associated with the positive feedback mechanisms between global warming and water vapor^17^. Furthermore, it can also be seen in Fig. 3a that upper tropospheric water vapor is primarily concentrated in tropical regions (30° S–30° N), which is associated with the fact that the tropics contribute to approximately 2/3 of the water vapor feedback in the upper troposphere^6^. Figures 3b represent the latitude–time evolution of specific humidity anomalies after removing the annual cycle and monthly variations, respectively. It can be observed that the water vapor anomalies were primarily negative before the twenty-first century, while a positive response became more dominant after the twenty-first century. This may be attributed to the upward trend in water vapor anomalies under the influence of global warming, which results in specific humidity data before the twenty-first century generally remained below average, while data after the twenty-first century are almost always above average. Figure 3c depicts the latitude–time evolution of specific humidity anomalies data after filtering, from which we can also observe similar spatial characteristics of water vapor anomalies variations. In addition, we observe a notably strong positive response around the years 1972–73 (Strong El Niño), 1982–83 (Very Strong El Niño), 1987–88 (Strong El Niño), 1997–98 (Strong El Niño), 2010–11 (Strong La Niña), 2015–16 (Very Strong El Niño), and 2020–21 (Moderate La Niña). These years are almost all associated with strong ENSO events, indicating a significant impact of strong ENSO events on the upper tropospheric water vapor anomalies.

**Figure 4:**
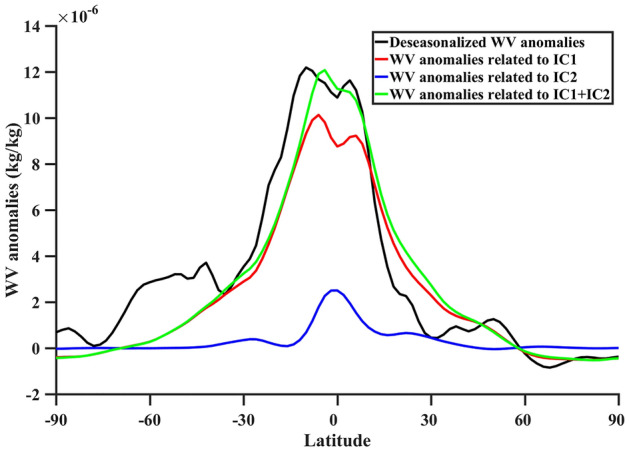
The latitude distribution of the deseasonalized water vapor (WV) anomalies (black) and of the water vapor anomalies related to the global warming pattern (red), ENSO pattern (blue) and global warming plus ENSO pattern (green) derived from ICA.

The study of Shine et al.^46^ points out that from a radiometric perspective, percentage variations in water vapor rather than absolute variations are more relevant to water vapor feedback. The findings of our study indicate that 53% of the global interannual variations in water vapor can be attributed to global warming, while 33% of the global interannual variations in water vapor can be explained by ENSO. In addition, the remaining 14% of the water vapor change may be related to water vapor transport caused by wind, seasonal changes, and other factors^47^. Moreover, to further investigate the latitude characteristics of water vapor and analyze the contributions of global warming and ENSO to water vapor, the latitude distribution of the deseasonalized water vapor anomalies, global warming pattern (IC1), ENSO pattern (IC2), and global warming plus ENSO pattern were generated (Fig. 4). From the results, it can be observed that the water vapor anomalies associated with global warming pattern (IC1) largely capture variations of water vapor in the upper troposphere. This is consistent with the fact that the IC1 pattern explains 53% of the global interannual variations in water vapor, indicating that global warming pattern is a primary factor driving variations in water vapor in the upper troposphere. Furthermore, we observed that the influence of ENSO pattern (IC2) on water vapor anomalies in the upper troposphere is primarily concentrated in tropical regions. This may be linked to the fact that ENSO originates in the tropical Pacific and tropical convection^4,48^. Moreover, we combined the contributions of global warming pattern (IC1) and ENSO pattern (IC2) on water vapor in the upper troposphere. We found that, except for a few areas (e.g., 30° S–60° S), the combined results are closely consistent with the upper tropospheric water vapor anomalies. The changes in water vapor result from the influence of multiple factors, while IC1 and IC2 respectively representing global warming and ENSO only capture the impacts of these two factors on water vapor. Besides, global warming is a steady, globally uniform forcing phenomenon, whereas the impact of ENSO on water vapor is primarily existed in tropical regions. Thus, the mentioned reasons could result in differences in the relationship between the deseasonalized water vapor anomalies and IC1, IC2, and IC1 + IC2 in certain regions. Therefore, we believe that global warming and ENSO patterns are significant factors contributing to variations in the upper tropospheric water vapor.

### The response of the upper tropospheric water vapor to global warming

**Figure 5:**
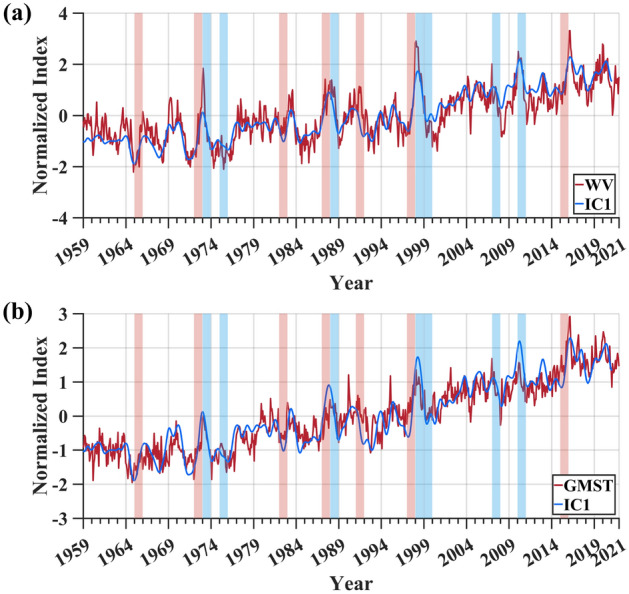
Correlation analysis of the time series of IC1 and the upper tropospheric water vapor. (**a**) the time series of the upper tropospheric water vapor anomalies (red line) and IC1 (blue line) extracted by the strategy of ICA combined with the non-parametric optimal dimension selection; (**b**) the time series of GMST (red line) and IC1 (blue line). The time series in this paper are normalized to overcome the effect of amplitude uncertainty in separating components with ICA.

To quantitatively analyze the relationship between upper tropospheric water vapor variations and global warming, the time series of the first independent component (IC1) of upper tropospheric specific humidity anomalies is obtained by employing the strategy of ICA combined with the non-parametric optimal dimension selection, as shown in Fig. 5.

The results of Independent Component Analysis indicate that the pattern of IC1 accounts for 53% of the global interannual variations in water vapor. The correlation coefficient between the time series of IC1 and the upper tropospheric water vapor anomalies is 0.87 (Fig. 5a). At the same time, it exhibits a significant correlation with GMST indicator representing the global warming signal, with a correlation coefficient as high as 0.91 (Fig. 5b) with a lag of 11 months (i.e., the maximum correlation coefficient with the GMST indicator is achieved after 11 months). As global warming is the response to a steady, globally uniform forcing^23^, altering convection is slower. Therefore, it requires a longer time to impact upper tropospheric water vapor compared to ENSO. The above results indicate that the pattern of IC1 captures the main features of water vapor variations, and a primary factor leading to upper tropospheric water vapor variations is global warming. Furthermore, we observed that there is usually a local peak or minimum in both water vapor anomalies and GMST during strong El Niño or La Niña years (highlighted in red and blue backgrounds in the figures). This suggests that strong ENSO events may have a significant impact on both global warming and water vapor anomalies. A possible explanation is that ENSO alters tropical convection, leading to variations in upper tropospheric water vapor. Subsequently, under the influence of the positive feedback mechanism of water vapor, it contributes to the variations in greenhouse gases, thereby influencing global warming. The correlation coefficient between the global warming pattern and the upper tropospheric water vapor anomalies is 0.87. Moreover, the global warming pattern also explains as much as 53% of the global interannual variations in water vapor, indicating that the upper tropospheric water vapor change is highly dependent on global warming. This correlation result also supports the results of Allan et al. (2022) that at higher altitudes in the troposphere, the global mean water vapor responds more strongly to temperature changes. This may be related to the following reason. According to the Clausius–Clapeyron equation, atmospheric water vapor increases with rising temperatures^49^. Therefore, the atmospheric water vapor content will increase under the influence of global warming. Water vapor is also a greenhouse gas, and with the increase in greenhouse gases, the amount of longwave radiation reaching the Earth's surface also intensifies, further exacerbating global warming^23^. Consequently, this leads to a heightened correlation between global warming patterns and water vapor in the upper troposphere.

**Figure 6:**
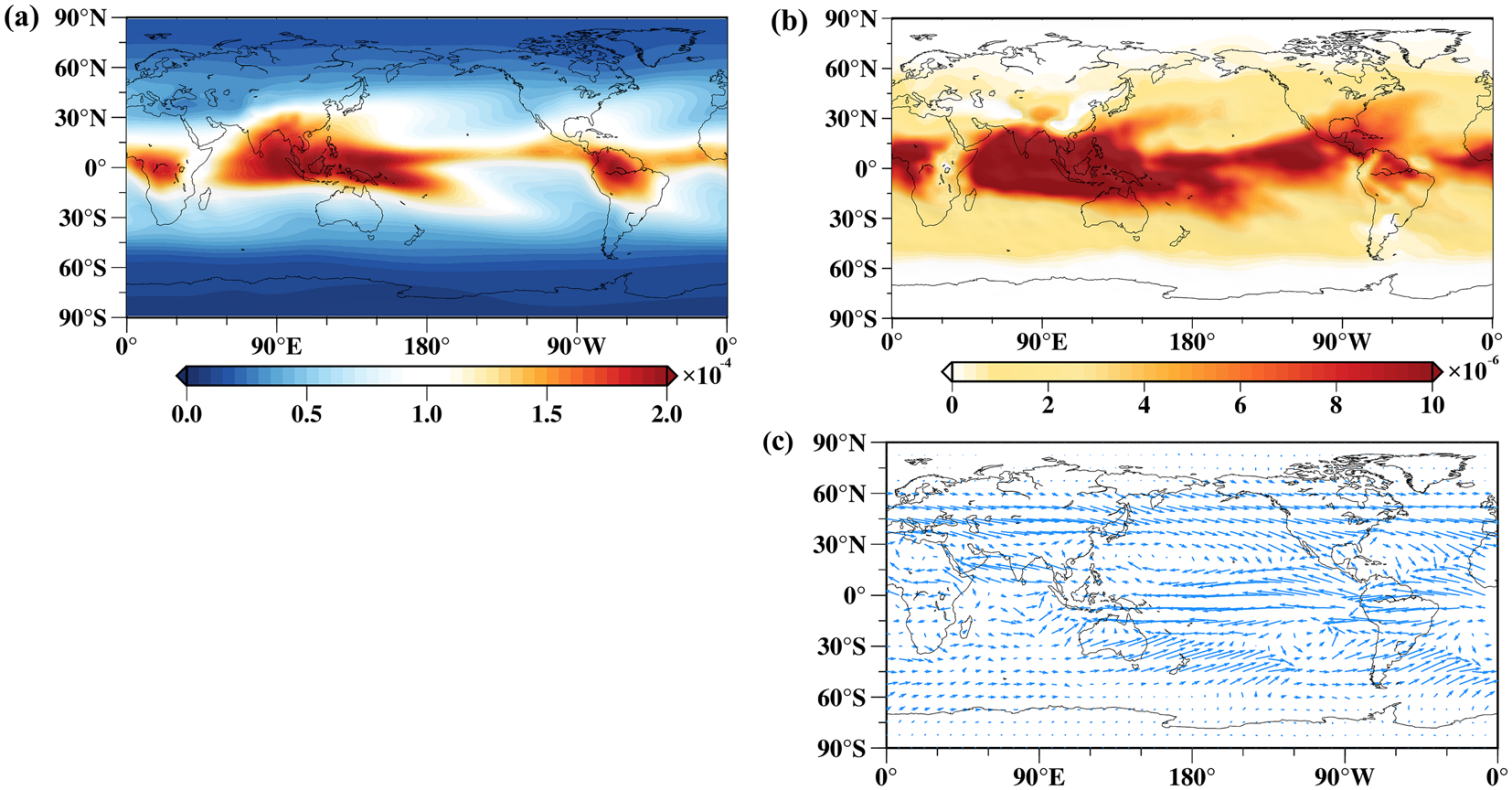
Analysis of the spatial relationship between global warming and the upper tropospheric water vapor. (**a**) The 63-year mean of upper tropospheric specific humidity at the 250 hPa pressure level (Units: kg/kg); (**b**) the spatial pattern of IC1 (Units: kg/kg). (**c**) The 63-year mean of the vertically integrated water vapor fluxes at global grid points (Units: mg/s kg).

Global warming intensifies the water vapor transport between the ocean and land^18^. To analyze the spatial response of upper tropospheric water vapor to global warming, Fig. 6 depicts the global distribution of the 63-year long-term mean of upper tropospheric specific humidity (Fig. 6a) and the spatial pattern of IC1 (Fig. 6b). The results of the global distribution of long-term mean of upper tropospheric specific humidity (Fig. 6a) are consistent with the water vapor distribution obtained by Takahashi et al. ^50^ and Tian et al. ^51^. The distribution of upper tropospheric water vapor shown in Fig. 6a is very close to the spatial pattern of IC1 (global warming pattern), indicating that the upper tropospheric water vapor is significantly influenced by the global warming pattern. Wang et al.^18^ pointed out that the exchange of water vapor between oceans and land near South America, Africa, and Australia is more sensitive under global warming conditions, and the spatial pattern of IC1 (Fig. 6b) also illustrates this spatial characteristic. The Intergovernmental Panel on Climate Change (IPCC) has indicated that global warming will further strengthen the large-scale atmospheric circulation, leading to significant changes in the global and regional water cycles^52^. This will intensify the transport of water vapor between oceans and land, consequently increasing the likelihood of extreme precipitation events and floods^18^. To investigate the relationship between global warming patterns and water vapor transport, this study employs the water vapor flux to depict the exchange of water vapor between the oceans and the land (Fig. 6c). The results indicate that the region of higher water vapor flux is approximately the same as the region affected by global warming (60° N–60° S). Additionally, regions such as Southeast Asia, and the lower part of North America experience pronounced warming (Fig. 6b), while at the same time, water vapor fluxes are also significant where land borders the ocean near these locations. Simultaneously, there is a significant water vapor flux in the mid-latitude region of the southern hemisphere pacific (30° S–60° S), which may be associated with the expansion of the Hadley circulation in the southern hemisphere, influenced by global warming (IPCC). Above results indirectly validate that under the influence of global warming significant changes in the global and regional hydrological cycles are occurring, and that water vapor exchange between oceans and land will also be intensified^18,52,53^.

### The response of the upper tropospheric water vapor to ENSO

**Figure 7:**
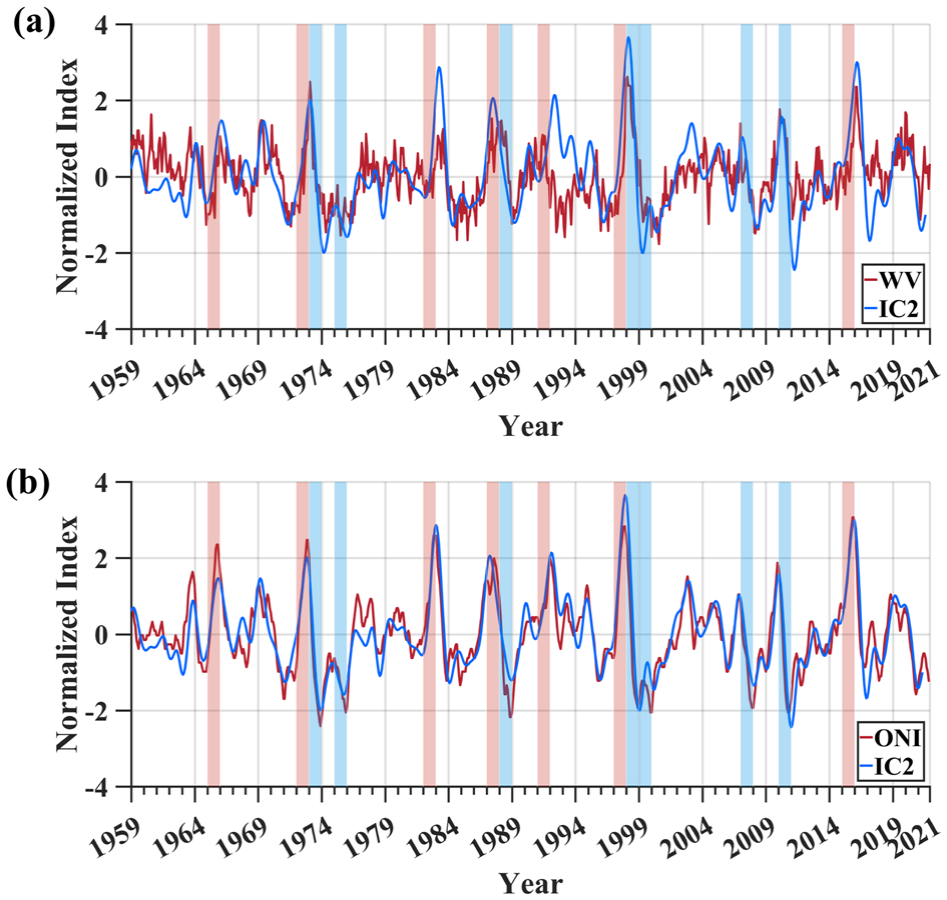
Correlation analysis of the time series of IC2 and the upper tropospheric water vapor. (**a**) The time series of the upper tropospheric water vapor anomalies after removal of linear trends (red line) at the 250 hPa pressure level and IC2 (blue line) extracted by the strategy of ICA combined with the non-parametric optimal dimension selection; (**b**) the time series of ONI (red line) and IC2 (blue line). The time series in this paper are normalized to overcome the effect of amplitude uncertainty in separating components with ICA.

ENSO exhibits a strong influence on the interannual anomalies of tropical upper tropospheric water vapor^4^. Therefore, to quantitatively analyze the response of upper tropospheric water vapor to ENSO, the time series of another independent component (IC2) of upper tropospheric specific humidity anomalies is also obtained, as shown in Fig. 7.

According to the results of independent component analysis, the pattern of IC2 accounts for 33% of the global interannual variations in water vapor. Due to the influence of the linear trend caused by global warming on upper tropospheric water vapor anomalies, the upper tropospheric water vapor anomalies were detrended according to the method of Lu et al. ^16^. The correlation coefficient between the time series of upper tropospheric water vapor anomalies after removing the linear trend and IC2 is 0.61 (Fig. 7a). Additionally, with an 8 month lag, a significant correlation was found between ONI and a correlation coefficient as high as 0.91 (Fig. 7b). The above results indicate that the pattern of IC2 also captures the characteristics of water vapor variations and that the other major factor contributing to water vapor variations in the upper troposphere is ENSO.

**Figure 8:**
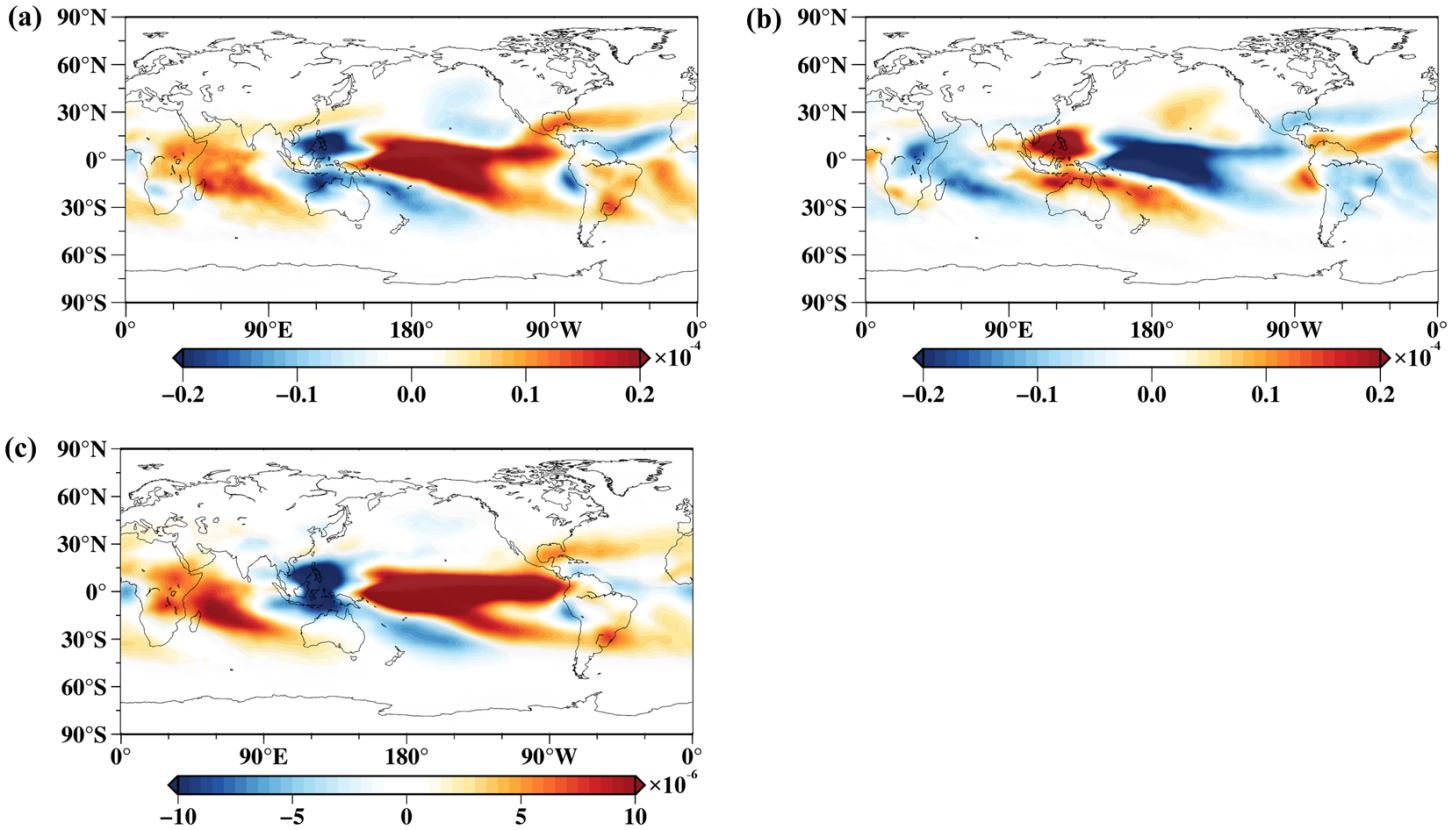
Analysis of the spatial relationship between ENSO and the upper tropospheric water vapor (Units: kg/kg). (**a**) The El Niño-year mean of upper tropospheric specific humidity at the DJF months at the 250 hPa pressure level; (**b**) the La Niña-year mean of upper tropospheric specific humidity at the DJF months at the 250 hPa pressure level; (**c**) the spatial pattern of IC2.

ENSO usually peaks during winter, which is also the period when ENSO has the strongest impact on water vapor^4^. Therefore, to analyze the spatial response of upper tropospheric water vapor to ENSO, Fig. 8 illustrates the El Niño-year mean of upper tropospheric specific humidity during the DJF months (Fig. 8a), the La Niña-year mean of upper tropospheric specific humidity at the DJF months (Fig. 8b), and the spatial pattern of IC2 (Fig. 8c). The spatial pattern results indicate that the global distribution of upper tropospheric specific humidity is mainly positive response during El Niño events, while the global distribution of upper tropospheric specific humidity is significant negative response during La Niña events. This result is consistent with the results of Saha et al. ^4^. Additionally, we found that the El Niño-year mean of upper tropospheric specific humidity during the DJF months (Fig. 8a) is very consistent with the spatial pattern of IC2, and both of them exhibit the classic ENSO pattern^48^, indicating that the upper tropospheric water vapor is affected by the El Niño pattern during El Niño events, resulting in a consistent spatial response. Generally, ENSO has a positive response on upper tropospheric water vapor under the influence of El Niño, leading to relatively moist conditions. Similarly, ENSO has a negative response on upper tropospheric water vapor during La Niña years, resulting in relatively dry conditions in the upper troposphere. In addition, relatively dry patches (blue patches) appeared next to warm pool under El Niño conditions (Fig. 8a, c) consistent with the results of Zhu et al. ^47^, which may be related to convection in the tropics.

**Figure 9:**
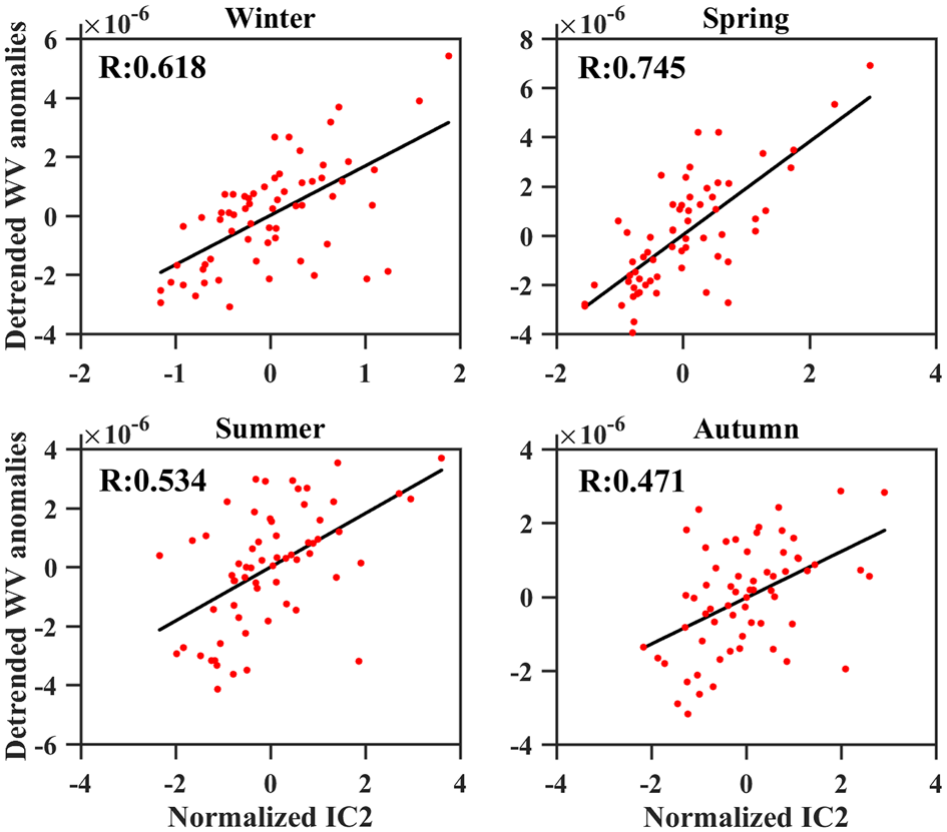
Scatter plots of water vapor anomalies versus normalized IC2 (ENSO pattern) in winter (DJF = December, January, and February), spring (MAM = March, April and May), summer (JJA = June, July and August), and autumn (SON = September, October, November) (Units: kg/kg). The values in the upper left corner are the correlation coefficients, where the p-values for correlation coefficients R in winter, spring, summer, and autumn are all less than 0.01.

ENSO exhibits seasonality^54^. Scatter plots of detrended water vapor anomalies and normalized IC2 (ENSO pattern) in winter, spring, summer, and autumn were generated to investigate the impact of ENSO on the upper tropospheric water vapor during different seasons (Fig. 9). The correlation coefficients between the upper tropospheric water vapor anomalies and normalized IC2 in winter, spring, summer, and autumn are 0.62, 0.74, 0.53, and 0.47, respectively. The results indicate that the correlation coefficient between upper tropospheric water vapor and ENSO pattern is higher during winter and spring, while it is lower during summer and autumn. This may be attributed to the seasonality of ENSO, as it peaks during DJF, but it takes months for the effects of ENSO to propagate to the upper tropospheric water vapor. The above results indicate that the influence extent of ENSO on upper tropospheric water vapor varies with the changing seasons.

## Conclusion

Understanding the characteristic variations of upper tropospheric water vapor and identifying the controlling factors influencing them are crucial for comprehending global climate change. Although many studies have indicated the impact of ENSO and global warming on water vapor, the relationship of ENSO and global warming on the variations in upper tropospheric water vapor is not yet fully understood. ERA5 specific humidity data from the European Centre for Medium-Range Weather Forecasts (ECMWF) is a product of combining data assimilation with weather forecast modelling system, the accuracy of which relies on data assimilation methods and the stability and precision of observational systems. On this basis, we extracted the temporal and spatial patterns of ENSO and global warming from January 1959 to December 2021 by employing the strategy of ICA combined with the non-parametric optimal dimension selection to address the problem of the non-Gaussian distribution characteristics of climate time series and the uncertainty in the output dimension selection of ICA. According to the results of global warming and ENSO patterns, the characteristics of water vapor variations in the upper troposphere and response to global warming and ENSO are revealed, providing crucial scientific insights for understanding global climate change. The main findings are as follows.

We analyzed the variations characteristics of water vapor in the upper troposphere and identified the factors affecting water vapor. There is an obvious seasonal variability of water vapor in the upper troposphere, which is usually the highest in summer and the lowest in winter. In addition, there is a clear upward trend in the time series of specific humidity anomalies, which may be associated with global warming according to the Clausius–Clapeyron equation. The water vapor in the upper troposphere has obvious latitude characteristics. The upper tropospheric water vapor is mainly concentrated in the tropics (30° N–30° S), and under the influence of the positive feedback mechanism of global warming, the longer the time scale, the greater the intensity and extent of the water vapor response in the upper troposphere. Most importantly, global warming and ENSO are crucial factors contributing to variations in upper tropospheric water vapor, with water vapor contributions due to global warming and ENSO patterns accounting for 86% of the global interannual variations in water vapor. In addition, the remaining 14% of the water vapor change may be associated with water vapor transport caused by wind, seasonal changes, and other factors. In particular, peaks in water vapor anomalies are associated with years of strong El Niño, while minimum values are associated with years of strong La Niña. The response of upper tropospheric water vapor to both global warming and ENSO takes a certain amount of time (i.e., it takes 11 months of propagation for the global warming pattern to have an impact on upper tropospheric water vapor, while ENSO takes 8 months of propagation). Global warming is the response to a steady, globally uniform forcing, thus taking a longer time compared to ENSO.

This study quantitatively analyzes the response relationship between variations in upper tropospheric water vapor and global warming. It is shown that not only does the global warming pattern (IC1) account for 53% of the global interannual variations in water vapor, but also a significant correlation with the upper tropospheric specific humidity anomalies is achieved. On the other hand, the spatial pattern of the global warming (IC1) is also very similar to the spatial pattern of the 63-year mean of upper tropospheric specific humidity. The above results indicate that global warming pattern (IC1) captures the majority of water vapor variations and is one of the primary factors driving variations in upper tropospheric water vapor. The correlation coefficient between the global warming pattern and the upper tropospheric water vapor anomalies is 0.87 and the global warming pattern also explains as much as 53% of the global interannual variations in water vapor, indicating that the upper tropospheric water vapor change is highly dependent on global warming. This may be related to the following reason. According to the Clausius–Clapeyron equation, atmospheric water vapor increases with rising temperatures^49^. Therefore, the atmospheric water vapor content will increase under the influence of global warming. Water vapor is also a greenhouse gas, and with the increase in greenhouse gases, the amount of longwave radiation reaching the Earth's surface also intensifies, further exacerbating global warming^23^. Consequently, this leads to a heightened correlation between global warming patterns and water vapor in the upper troposphere. Under the influence of global warming, there also will be significant changes in the water cycle at the global and regional scales, as well as an intensification of water vapor transport between the oceans and the land. Furthermore, both upper tropospheric water vapor anomalies and GMST achieve local peaks or minima during strong El Niño or strong La Niña years. This is related to the fact that strong ENSO events may have a significant impact on water vapor variations. Under the influence of the positive feedback mechanism of water vapor, it contributes to the variations of greenhouse gases, thereby influencing global warming.

The study also quantitatively analyzed the relationship between upper tropospheric water vapor variations and ENSO. It is shown that the ENSO pattern (IC2) accounts for 33% of the global interannual variations in water vapor and also has a strong correlation with the upper tropospheric specific humidity anomalies. Without considering the phase, the spatial pattern of the ENSO (IC2) is very close to the distribution of ENSO-year mean of upper tropospheric specific humidity during the DJF months. The above results suggest that ENSO captures some of the water vapor variations and is also one of the main factors contributing to water vapor variations in the upper troposphere. The results also show that the influence extent of ENSO on upper tropospheric water vapor varies with the changing seasons. During El Niño events, the distribution of global upper tropospheric water vapor is characterized by mainly positive anomalies, while during La Niña events, it is characterized by mainly negative anomalies. Additionally, relatively dry patches (blue patches) were observed near the warm pool during El Niño years, which is likely associated with tropical convection.

## Data Availability

ERA5 reanalysis data can be obtained from https://cds.climate.copernicus.eu/cdsapp#!/dataset/reanalysis-era5-pressure-levels-monthly-means?tab=form. The global mean surface temperature (GMST) indicator can be obtained from https://climate.nasa.gov/vital-signs/global-temperature/. Oceanic Niño Index (ONI) can be obtained from https://psl.noaa.gov/data/correlation/oni.data. The vertically integrated water vapor fluxes at global grid points can be obtained from https://www.geodata.cn/data/datadetails.html?dataguid=996261963&docid=22000.

## References

[1] Dessler AE, Zhang Z, Yang P (2008). Water-vapor climate feedback inferred from climate fluctuations, 2003–2008. Geophys. Res. Lett..

[2] Allan RP, Shine KP, Slingo A, Pamment JA (1999). The dependence of clear-sky outgoing long-wave radiation on surface temperature and relative humidity. Q. J. R. Meteorol. Soc..

[3] Colman R, Soden BJ (2021). Water vapor and lapse rate feedbacks in the climate system. Rev. Mod. Phys..

[4] Saha J, Price C, Plotnik T, Guha A (2022). Impact of the El Niño-Southern Oscillation on upper-tropospheric water vapor. Atmos. Res..

[5] Kim H-M, Zhou Y, Alexander MA (2019). Changes in atmospheric rivers and moisture transport over the Northeast Pacific and western North America in response to ENSO diversity. Clim. Dyn..

[6] Held IM, Soden BJ (2000). Water vapor feedback and global warming. Annu. Rev. Energy. Environ..

[7] Wang J, Cole HL, Carlson DJ (2001). Water vapor variability in the tropical western pacific from 20-year radiosonde data. Adv. Atmos. Sci..

[8] Hansen, J. *et al.* Climate sensitivity: Analysis of feedback mechanisms. In *Climate Processes and Climate Sensitivity* 130–163 (American Geophysical Union (AGU), 1984). 10.1029/GM029p0130.

[9] Paltridge G, Arking A, Pook M (2009). Trends in middle- and upper-level tropospheric humidity from NCEP reanalysis data. Theor. Appl. Climatol..

[10] Vergados P, Mannucci AJ, Ao CO, Fetzer EJ (2016). Using GPS radio occultations to infer the water vapor feedback. Geophys. Res. Lett..

[11] Allan RP (2012). The role of water vapour in earth’s energy flows. Surv. Geophys..

[12] Allan RP, Willett KM, John VO, Trent T (2022). Global changes in water vapor 1979–2020. J. Geophys. Res.: Atmos..

[13] Gettelman A, Fu Q (2008). Observed and simulated upper-tropospheric water vapor feedback. J. Clim..

[14] Chen C-T, Roeckner E, Soden BJ (1996). A comparison of satellite observations and model simulations of column-integrated moisture and upper-tropospheric humidity. J. Clim..

[15] Trenberth KE, Fasullo J, Smith L (2005). Trends and variability in column-integrated atmospheric water vapor. Clim. Dyn..

[16] Lu J (2020). Analysis of factors influencing tropical lower stratospheric water vapor during 1980–2017. NPJ Clim. Atmos. Sci..

[17] Patel VK, Kuttippurath J (2023). Increase in tropospheric water vapor amplifies global warming and climate change. Ocean-Land-Atmos. Res..

[18] Wang J (2023). Atmospheric water vapor transport between ocean and land under climate warming. J. Clim..

[19] Lindzen RS (1990). Some coolness concerning global warming. Bull. Am. Meteorol. Soc..

[20] Pierrehumbert RT (1995). Thermostats, radiator fins, and the local runaway greenhouse. J. Atmos. Sci..

[21] Rind D (1991). Positive water vapour feedback in climate models confirmed by satellite data. Nature.

[22] Dickinson, R. *et al. Climate Change 1995: The Science of Climate Change. Contribution of WG1 to the Second Assessment Report of the IPCC* (Cambridge University Press, 1996).

[23] Hall A, Manabe S (1999). The role of water vapor feedback in unperturbed climate variability and global warming. J. Clim..

[24] Hsieh WW (2004). Nonlinear multivariate and time series analysis by neural network methods. Rev. Geophys..

[25] Burgers G, Stephenson DB (1999). The, “normality” of El Niño. Geophys. Res. Lett..

[26] An S-I, Wang B (2000). Interdecadal change of the structure of the ENSO mode and its impact on the ENSO frequency. J. Clim..

[27] Mudelsee M (2019). Trend analysis of climate time series: A review of methods. Earth-Sci. Rev..

[28] Jutten C, Herault J (1991). Blind separation of sources, part I: An adaptive algorithm based on neuromimetic architecture. Signal Process..

[29] Comon P (1994). Independent component analysis, A new concept?. Signal Process..

[30] Koch I, Naito K (2007). Dimension selection for feature selection and dimension reduction with principal and independent component analysis. Neural Comput..

[31] Blunden J, Boyer T, Bartow-Gillies E (2023). State of the climate in 2022. Bull. Am. Meteorol. Soc..

[32] Shi L (2022). Assessing the consistency of satellite-derived upper tropospheric humidity measurements. Atmos. Meas. Tech..

[33] Tian B, Hearty T (2020). Estimating and removing the sampling biases of the AIRS Obs4MIPs V2 data. Earth Space Sci..

[34] Shao X (2023). Characterizing the tropospheric water vapor spatial variation and trend using 2007–2018 COSMIC radio occultation and ECMWF reanalysis data. Atmos. Chem. Phys..

[35] Hersbach, H. *et al.**ERA Report Series[J]*. https://www.ecmwf.int/en/forecasts/datasets/reanalysis-datasets/era-interim (2018).

[36] Hersbach H (2020). The ERA5 global reanalysis. Q. J. R. Meteorol. Soc..

[37] Trenberth KE, Stepaniak DP (2001). Indices of El Niño evolution. J. Clim..

[38] National Academies of Sciences, E. M. *et al. Review of the Draft Fourth National Climate Assessment*. (National Academies Press, 2018).

[39] Stocker T (2014). Climate Change 2013: The Physical Science Basis: Working Group I Contribution to the Fifth Assessment Report of the Intergovernmental Panel on Climate Change.

[40] Valipour M, Bateni SM, Jun C (2021). Global surface temperature: A new insight. Climate.

[41] Sun L, Lan Y (2021). Statistical downscaling of daily temperature and precipitation over China using deep learning neural models: Localization and comparison with other methods. Int. J. Climatol..

[42] Wang D, Qin Y, Xiao X, Zhang Z, Wu XE (2012). Niño and El Niño Modoki variability based on a new ocean reanalysis. Ocean Dyn..

[43] Chen Z (2024). The impact of global warming on ENSO from the perspective of objective signals. Atmos. Res..

[44] Chen Z, Li J, Luo J, Cao X (2018). A new strategy for extracting ENSO related signals in the troposphere and lower stratosphere from GNSS RO specific humidity observations. Remote Sens..

[45] Tian EW, Su H, Tian B, Jiang JH (2019). Interannual variations of water vapor in the tropical upper troposphere and the lower and middle stratosphere and their connections to ENSO and QBO. Atmos. Chem. Phys..

[46] Shine KP, Sinha A (1991). Sensitivity of the Earth’s climate to height-dependent changes in the water vapour mixing ratio. Nature.

[47] Zhu Y, Newell RE, Read WG (2000). Factors controlling upper-troposphere water vapor. J. Clim..

[48] Wallace JM (1998). On the structure and evolution of ENSO-related climate variability in the tropical Pacific: Lessons from TOGA. J. Geophys. Res. Oceans.

[49] Kininmonth W (2010). Clausius-clapeyron and the regulation of global warming. Fis. E.

[50] Takahashi H, Su H, Jiang JH (2016). Error analysis of upper tropospheric water vapor in CMIP5 models using “A-Train” satellite observations and reanalysis data. Clim. Dyn..

[51] Tian B (2013). Evaluating CMIP5 models using AIRS tropospheric air temperature and specific humidity climatology. J. Geophys. Res.: Atmos..

[52] Douville, H. *et al.**ipcc2021 Water Cycle Changes* In Climate Change 2021: The Physical Science Basis. Contribution of Working Group I to the Sixth Assessment Report of the Intergovernmental Panel on Climate Change. pp. 1055–1210 [Masson-Delmotte, V. *et al.* (eds.)] Cambridge University Press, Cambridge, United Kingdom and New York, NY, USA. 10.1017/9781009157896.010 (2023).

[53] Fernández-Alvarez JC (2023). Projected changes in atmospheric moisture transport contributions associated with climate warming in the North Atlantic. Nat. Commun..

[54] Rasmusson EM, Carpenter TH (1982). Variations in tropical sea surface temperature and surface wind fields associated with the southern oscillation/El Niño. Mon. Weather Rev..

